# Impact of COVID-19 – Experiences of 5th year medical students at the University of KwaZulu-Natal

**DOI:** 10.4102/safp.v64i1.5483

**Published:** 2022-06-14

**Authors:** Andrew J. Ross

**Affiliations:** 1 Discipline of Family Medicine, School of Nursing and Public Health, College of Health Sciences, University of KwaZulu-Natal, Durban, South Africa

**Keywords:** COVID-19, learning, qualitative, stress and anxiety, resilience

## Abstract

**Background:**

The global pandemic associated with coronavirus disease 2019 (COVID-19) had a considerable effect on higher education in South Africa, with online instruction replacing traditional lectures for many students. Medical students were required to vacate their residences in March 2020 but returned to campus in July 2020 to enable them to continue with clinical teaching and learning. The aim of this study was to understand the learning experiences of 5th year medical students at the University of KwaZulu-Natal (UKZN) during 2020.

**Methods:**

This was a qualitative study conducted via Zoom in December 2020 with 18 students in four focus group discussions and four semi-structured interviews. These were all facilitated by an independent researcher with experience in qualitative research. All the interviews were recorded, transcribed verbatim and analysed qualitatively through the identification of codes, categories and themes.

**Results:**

The following major themes emerged: A stressful and at times an overwhelming year, mental health issues, developing strategies to cope, and issues that related to teaching and learning.

**Conclusion:**

The disruptions caused by COVID-19, the lockdown, a condensed academic programme and uncertainty about their competency resulted in high levels of anxiety and stress among medical students. Participants highlighted strategies that had helped them to cope with the isolation and academic pressures. Given the large volume of work, careful thought needs to be given to what should be taught and how it should be taught to ensure that graduates have the competencies they need to practise.

## Background

The global pandemic associated with coronavirus disease 2019 (COVID-19) had a considerable effect on higher education in South Africa and globally.^1^ For all students in tertiary education, 2020 was a challenging year, but for the 4th–6th year medical students at the University of KwaZulu-Natal (UKZN) it was a tumultuous year full of disruptions. Students started their university blocks early in January 2020, and shortly thereafter went on a three-week strike as many of their colleagues were threatened with financial exclusion due to the non-payment of their fees. Once agreement was reached with the university management not to exclude students for financial reasons, they returned to their clinical sites for 4 weeks prior to the national lockdown announced by President Ramaphosa on 20 March 2021. Following the lockdown and the restrictions on face-to-face/in-person teaching, UKZN was forced to move onto online learning platforms.^2^ Unfortunately, this online teaching was delayed for 12 weeks at UKZN as staff needed to transition to the new platform, and the university wanted to ensure that every student had access to a suitable device and sufficient data so that ‘no student was left behind’.

Similar to most of the medical schools in South Africa, the MBChB programme at UKZN is a 6-year programme. Years 1–3 are preclinical and years 4–6 are clinical, where theoretical teaching is combined with clinical instruction. Due to the COVID-19 disruptions students in their clinical years received 6-weeks online, Zoom-based teaching from 8 June 2020 to 17 July 2020 and resumed clinical teaching on 20 July 2020. All lectures were recorded and uploaded onto Moodle (learning platform used by UKZN) and AMBOS, an online international learning platform management system, was made available to all medical students at UKZN. Students returning to the clinical platform had a week of orientation, followed by a week of exams to finish off the first block, and then five times four-week blocks (5 × 4 weeks) from 20 July 2020 to 15 December 2020, with supplementary examinations being held in January 2021.

The return to campus meant that students had access to reliable Wi-Fi, electricity and an environment conducive to learning. Each of the clinical blocks were structured differently by the academic departments, with varying levels of clinical exposure. All students were provided with appropriate personal protective equipment and hand sanitizers, and clinical departments were asked not to rotate students through COVID-19 wards or allow them to see patients under investigation (PUI’s) for COVID-19.

In the 5th year family medicine block, students spent alternate days on the clinical platform at a district hospital. On the days that they were not allocated to the hospital, students had a 2-h Zoom-based tutorial with a family physician and spent the rest of the day working on their portfolio tasks, reading around patients they had seen and preparing for the end of block examinations. Assessments were held during the last week of the block. Little is known about the learning experience of 5th year medical students at UKZN during 2020. This study aimed to understand how their learning experiences had been affected by the changing learning environment due to the COVID-19 restrictions.

## Methods

This was a qualitative study conducted via Zoom in December 2020. All 5th year medical students at UKZN (*n* = 254) were contacted via a personal e-mail in December 2020 to request their willingness to participate in a focus group discussion (FGD) or a semi-structured interview (SSI) to share their experiences of learning during the year. Details of the research were emailed to the 18 who indicated a willingness to participate, which resulted in four Zoom-based FGDs with three to four participants per group and four SSIs that were facilitated by an independent researcher with experience in qualitative research between 8 December 2020 and 14 December 2020. An interview guide was used in both the SSIs and the FGDs to ensure that all groups were asked the same question(s).

After the introductions and an explanation of the purpose of the research, all students gave written consent to participate via the chat box on Zoom, and were asked ‘Tell me about your learning experiences during this year with all the challenges associated with COVID-19’ with summaries and clarification questions asked by the facilitator. Probing questions about the Zoom teaching, clinical exposure and assessments were asked if these were not covered. In addition, if not spontaneously raised, students were asked about their strategies to cope with the challenges they faced as well as any resources provided by UKZN. The interviews lasted 40–80 min and were recorded, transcribed verbatim and analysed qualitatively by identifying codes, categories and themes. This was done by the author, who is the 5th year family medicine coordinator, in consultation with colleagues. Data from multiple interviews ensured triangulation of the finding.

### Ethical considerations

Gate keeper permission was given by the Registrar of the University of KwaZulu-Natal, and ethical clearance to conduct this study was obtained from the University of KwaZulu-Natal, Social Science Research Ethics Committee. (number: HSSREC/00002180/2020)

## Results

Four women participated in the SSIs, while seven men and eight women (one who also participated in the SSI) participated in the FGDs. The following major themes were identified:

- A stressful and at times overwhelming year.
- Mental health issues.
- Developing coping strategies.
- Teaching and learning.
- Grateful that the academic year was salvaged.

### Theme 1: A stressful and at times overwhelming year

The dominant theme to emerge from all the students was that 2020 had been an extremely stressful, and at times, an overwhelming year. They often used the words *stressful, challenging, tough, overwhelming, anxious* and *hectic* when describing their experiences of learning during the COVID-19 pandemic of 2020:

‘It’s been an incredibly, challenging year’. (Focus group discussion.’ [FGD] 3, TM, FGD 4, NN)

‘… it’s been a very, very tough year’. (FGD 2 SB)

‘… it was very overwhelming.’ (FGD 4, Zama)

‘… in literally every single block, I felt so overwhelmed.’ Semi-structured interview [SSI] 4, LZ)

‘Everyone’s been stressed, everyone’s anxious and then we have studying on top of that, and that’s been a bit hectic, you know’. (FGD 3, DD)

Many factors contributed to this, including poor communication from the university, uncertainty about their future, sickness and death at home, shortened blocks with a heavy workload and the fear of taking COVID-19 home.

Students were critical of the ‘radio silence’ (FGD 1, LZ) from the university, and receiving ‘no email, no SMS, no communication, no nothing’ (FGD 1, LZ) from lockdown in March 2020 until June 2020. This lack of communication created stress and uncertainty for them during the ‘three months that we were sitting at home’ (FGD 1, LZ). Although the situation was unprecedented, they were critical that the university had not made more effort to clearly communicate a plan as ‘… we don’t just don’t know what’s happening. I think that the university actually created more chaos than it was able to curb [*due to the lack of communication*]’ (FGD 1, LZ).

The students felt that ‘Even if they told us in March that you’re only coming back in November, at least there would have been a certainty and an ability to plan’ (SSI 4, LZ). The lockdown, the lack of communication from the university and the resulting uncertainly triggered anxiety, and some students felt that their ‘career was slipping away’ (FGD 1, ET). These feelings were compounded by tragedy in the family: ‘I lost two of my uncles in two weeks’ (FGD 2, SM); financial stress ‘… the biggest challenge was finances’ (FGD 4, SM), ‘due to COVID everyone was at home, not working’ (FGD 4, SM), and no space/time to deal with the emotions associated with all these changes as ‘you can’t even really deal with emotions. You can’t mourn, you try to avoid it’ (FGD 2, SM). In addition, there was considerable academic pressure to pass and at times an overwhelming ‘fear of failure’ (FGD 2, SM), which was compounded by the shortened blocks.

For many, it was also an isolating and socially alienating year, with little contact with friends and other support structures.

‘[*T*]his year has been the most alienating year that I’ve ever experienced at university. I had to deactivate all my social media because I just could not split the attention between Facebook and Instagram and Twitter and my school. I had to cut that off, I just did not have time. For me, activism is my outlet. I had to stop that or else I would not cope with the school’. (FGD 1, LZ)

When the clinical blocks did start, those students staying at home, particularly with family members or relatives who had comorbidities, were anxious that they might ‘bring home a virus’ (FGD 3, DD). For some students, this fear was so incapacitating that it prevented them from studying and fully participating on the clinical platform when they did have the opportunity.

‘For me, it’s scary, especially because I’m coming home to my dad who does have co-morbidities and … the doctors expect us to go up to patients and just act like nothing’s wrong and think that the mask is going to protect us’. (SSI 2, YS)

### Theme 2: Mental health issues

Students felt ill-prepared to deal with the absence of communication from the university and new online learning environment, and felt that their mental well-being had been completely ignored, despite the institution knowing the amount of theoretical and practical work that they would be required to complete during the year. Their perception was that they received little support from staff or management about how to cope with the changing circumstances, despite their learning to be part of a discipline where mental health is regarded as an integral part of well-being:

‘[N]obody was focusing on mental health and nobody is helping you with that … it’s to school, school, school, academics, and I feel like certain aspects were neglected, somehow, somewhere … it’s like you’re being desensitized to the human in you’. (FGD 4, NN)

‘I felt that UKZN has really, really failed in terms of being able to take care of the mental health aspect’ (FGD 1, LZ**)**

and that

‘there was not a lot of psychological support that the University offers. They could have done more, seriously, because the students were really, really going through the most this year’. (FGD 4, LZ)

‘This year, my anxiety was like through the roof … I’m anxious as soon as the block starts … I’m anxious about studying, … I’m anxious about opening a book and studying and it’s never ever been like this’. (SSI 1, DK)

‘I don’t feel stronger. I feel completely broken. I feel completely lost. I don’t feel good. I feel like at this point I’m just doing this just to get through the next exam, the next exam and the next exam’. (FGD 1, DC)

However, the students also recognised that ‘medical school it’s stressful beside COVID-19’ (FGD 1, MX) and that:

‘[*Y*]ou need to know what helps you to cope with stressful situations, as we know that everyone reacts differently to stressful situations. … the most important thing is that you need to know where and how to get help’. (FGD 1, MX)

and learn to be resilient.

### Theme 3: Developing coping strategies

Despite the challenges of the year and the associated stress and uncertainty, students recognised that it was important for them to learn to look after their own physical and mental health. They recognised the need to learn from these experiences, to adapt and find strategies to enable them to cope with the changing circumstances. The students felt there was ‘so much to learn from 2020, there was COVID, so we had to learn to adapt’ (FGD 3, TM). The experiences of 2020 ‘brought a lot of change into my life, including self-growth, and academic growth’ (FGD 1, ET). While ‘it’s been a challenging year … I think it’s brought some great experiences and new ideas in many of us’ (FGD 1, MX). For some students:

‘[*I*]t pushed me to do better and it allowed for growth and introspection … I think I’ve learned a lot about myself in this year through all of the challenges’. (FGD 4, JK)

‘During COVID-19 it was important to know yourself … to understand yourself … what really helps you … if going to church helps you, you have you to go to church, … if going out with friends help you, you have to do that’. (FGD 1, MX)

Students talked about the importance of time management, keeping social connections, exercise and the need for ‘me time’. They emphasised the importance of ‘manag[ing] time to get the most out of it’ (SSI 4, LZ), studying effectively by ‘taking small breaks in between … deciding to do the important bits first, and if you have time go through to the extra bits’ (SSI 1, DK). They recognised the importance of keeping social connections with family and friends as ‘calling home and talking about it helped me a lot’ (FGD 4, SS).

‘For me, the thing that helps me the most in situations when I’m stressed is connecting with other people, especially friends and family – that was really helpful’. (FGD 1, MX**)**

Several students acknowledged the need to ensure that they had sufficient sleep and exercise, that staying physically healthy was an important part of their overall well-being to enable them to handle the uncertainty and stress associated with COVID-19.

‘Here in Pietermaritzburg there’s a mountain biking park that’s close by and they’ve got this really amazing hiking trail and we would go for a hike … that’s was one of the things, I think you just find ways to get your mind off things, but then you come back and you walk into your room and you’re like, there’s all of the books and now I need to start studying again’. (FGD 4, JK)

A number of students indicated that they took up new hobbies or pursued those that they had not had time to do, as that not only filled their time but gave them something enjoyable to focus on as:

‘[*L*]ockdown gave me time to work on myself, rather than academics … I started singing again and I started recording my own music … I did stuff like that to take my mind off academics’. (SSI 1, DK)

### Theme 4: Teaching and learning

Teaching and learning was impacted by five factors, these being (1) the initial six weeks of continuous content laden lectures delivered via Zoom, (2) the reduction of six-week clinical blocks to four weeks with limited clinical exposure, (3) end of block assessments every four weeks, (4) thoughts for the future, and (5) grateful that the academic year was salvaged.

#### Six weeks of lectures and content overload

While there were some challenges of access to the 6 weeks of online lectures during the lockdown, with stories of some students having to ‘climbing a mountain to get signal to watch the Zoom lectures’ (SSI 10, DK), and others having challenges with data as ‘some had already finished their data because it was received maybe three weeks prior’ (to the start of the online lectures) (FGD 3, NB), for most, ‘the data from UKZN was fine’ (FGD 2, ME**)**. Access and connectivity issues were resolved when students returned to campus, as they were able to utilise the facilities provided by the university. The major issue with the 6 weeks of lectures was the sheer volume of the work:

‘The lecture was 340 slides long, which was intimidating. So as a result, I took the conscious decision I’m not going to study this lecture, I decided that I’m not even going to stress myself developing an approach’. (FGD 1, LZ)

Students were given little academic direction, did not know what to focus on, and struggled with the disconnect between the theoretical teaching and the blocks, which made learning a challenge. Family responsibilities made it almost impossible for some to sit for 8 h a day, Monday to Friday, for six weeks:

‘In terms of our schoolwork, it’s been challenging, because for some of us, it’s almost impossible to read when you at home, because there’s a lot to do’ (FGD 4, LD) ‘… And culturally, there’s no way I can wake up in the morning in the house and have my 65-year-old aunt and my 58-year-old mom clean the house and I don’t participate in that’. (FG 1, LZ)

However, recording the lectures and placing them on Moodle meant that students were able to access them at a later date (for many students this meant accessing them when they got back to campus):

‘The recordings were on Moodle and were available. … honestly it was very much impossible to be present in every video, to listen and to study content. It was just, it was too much’. (FGD 4, SM)

‘But I was able to listen to the videos on Moodle when I came back to campus’. (FGD 3, TM)

#### Four weeks clinical blocks with limited clinical exposure

The four-week clinical blocks were an emergency response to ensure that the academic year was completed by the end of the calendar year. Although blocks are normally six weeks long in the 5th year, with the time lost due to the lockdown, they were reduced to five weeks (one week of lectures in June/July plus four weeks clinical attachment). The shortened four-week blocks were very demanding, as students were expected to be familiar with the content that had been covered during the six weeks of online lectures. In most of the blocks, the students went straight onto the clinical platform and exams were conducted during the last week of the block. This meant that there were 5 × 4 week blocks with no breaks, ‘no holidays [*and no time*] to see my family’ (FGD 2, ME) in between the blocks:

‘The four-week blocks, shame, we were dying’. (FGD 4, SM)

‘It’s literally like impossible to work (like that). It’s mentally not possible, you cannot like go straight from writing to studying full force again’. (SSI 2, YS)

‘It’s just suffering and more suffering, and all you have is a weekend after a month to pick up the pieces and get back to work’. (FGD 4, SM)

‘You’re in constant stress mode, you’re in constant fatigue. You know, it’s like literally chronic stress chronic, chronic fatigue. And I think the worst part is just having so much quantity, so much volume to study in such a small period of time’. (FGD 3, DD)

The students felt that the four-week block were incredibly challenging due to the perception that they now needed to fit ‘all of the content of six weeks into four weeks’ (FGD 2, ME), with the:

‘[*S*]ame resources that you were to use in a period of six weeks … and the approaches have not changed, nobody tells you how to now approach all this materials and how to utilize them productively, but in a shorter period of time’. (FGD 4, NN)

Students also felt that due to the way that the blocks were structured, they had missed out on clinical teaching and exposure, as ‘the clinical exposure was cut and it’s actually a loss to us’, (FGD 1, AW), as ‘correlating what you studied from a textbook to what you see in the wards is so much more helpful than just reading it in a textbook’ (FD 3, DD), as well as ward rounds and the opportunity to acquire the skills that they needed:

‘I don’t feel we had enough exposure. Like I’ve been thinking about next year, and oh my goodness, we’re doing final year, and we are not able to do some procedures, I mean, we’re just observing at times and doing in a bit here and there and it’s just not okay. For me, going to final year, it’s quite scary, so yeah’. (FGD 4, SS)

The lack of clinical exposure and cramming for exams made them feel incompetent. Despite the fact that they ‘passed after the four weeks and [*some*] got quite a good marks [*many of us*] don’t know what was going on and what we are doing’ (FGD 1, LZ). Even though ‘I’ve been doing really, really well … but at the end of it I don’t know anything’ (SSI 4, LZ) and ‘the marks that I’ve been getting are not a reflection of what I know’ (FGD 1, DC). The students expressed their concerns about not being adequately prepared for final year or equipped for internship:

‘I feel so unprepared for the future and going into internship. And the clinical exposure that we had this year was not enough because there’s certain things that we didn’t know and certain new concepts that we had to learn this year that you couldn’t learn’. (FGD 4, ZS)

They were also concerned that because of the gaps in their knowledge and skills due to the challenges that they faced during the lockdown, they would be branded as ‘useless’ (FGD 1, DC). They hoped that this branding would not happen and that the medical community would see this as an opportunity to ‘focus on developing young doctors and not just throw us aside saying, oh, this batch of 2020 5th years or this batch of 2021 6th years is going to be useless forever’ (FGD 1, DC).

#### Assessments every four weeks

Due to the constraints imposed by COVID-19, and concern for the safety of staff and students, the end of module assessment in 5th year Family Medicine module was changed. The clinical and face-to-face objective structured, clinical examinations (OSCE) was dropped, a greater emphasis was placed on submitting a portfolio of learning, the weighting of the multiple choice question (MCQ) exam was increased and an online Zoom-based OSCE examination (30%), where students reviewed exhibits (chest X-ray [CXR], electrocardiogram [ECG] etc.), did online consultations and had interactive discussions with examiners, was introduced. Students were encouraged to start the portfolio assignments during lockdown, and peer marking was introduced as an additional learning strategy, which they enjoyed and learnt from ‘as I had to mark other students work so, I learnt a lot. Yes, so I got to learn through some of the assignments’ (FGD 3, TM), and appreciated the staggered submission dates, which made it easier to keep up with the required work.

Although not ideal, as an OSCE is meant to allow student to ‘show how’ they complete a task, the online OSCE did allow for assessment in a structured and consistent manner. Students recognised that it was innovative, ‘very daring, and a risk to set up an exam that way’ (GFD 1, DC). Most students felt that the online OSCE ‘was done well, (and was) a good way of examining’ (FGD 1, DC), although there were challenges with technology that resulted in some students being ‘kicked out during the exam’ (FGD 2, SM).

In addition to the large volume of work and the shortened blocks, the content of the blocks was not reduced, and the students felt that the academic staff were not sympathetic or understanding and that they still expected them to perform at a very high level:

‘The academics, they demand so much from you in such a short space of time, and everything that has to do with you being human, emotions, mental health, it all comes to a standstill. … you’re still expected to perform and be competent with such little exposure … it got overwhelming, especially with everything that we had to do and everything that we had to cover …’. (FGD 4, NJ)

In addition, the students felt that with the shortened blocks, their brains were ‘so saturated that I couldn’t hold any more information’ (SSI 1, DK), that they were simply cramming for exams and not really learning anything. They were focused on ‘passing now’ (SSI 2, YS) but recognised ‘that it shouldn’t be I just want to pass, it should be you want to learn and help patients’ (SSI 2, YS). However, their reality was that ‘I’m mentally exhausted’ (SSI 1, DK), and expressed some uncertainty about whether or not:

‘I have absorbed a lot of information in this few months where we’ve had everything one after the other. It was more just studying to pass the exam, rather than having to learn something for life’. (FGD 2, SB)

#### Thoughts for the future

During the SSIs and FGD, the participants suggested a number of ways that teaching and learning under restricted conditions could be improved to ensure that they received all the relevant theoretical and practical instruction to be competent for their internship. These included planning and preparing for additional lockdowns, better communication between the university and students, training of staff to use the available technology to best effect, and a catch-up plan.

Participants recognised that COVID-19 was unlikely to be the last crises to be faced, and that blended teaching would in all likelihood continue into the future. They felt that the university needed to be more agile and innovative so that should the situation arise again, the university could:

‘[*A*]ct very swiftly if there is solid plan so that … everyone knows what needs to be done from day one and … include the students in the plan and not change the plan every one or two weeks’. (FGD 2, SM).

The students would:

‘[*L*]ike to see that the university is thinking ahead … like okay, we can do things as normal but if there’s a point when things are getting tough – we switch directly to online learning. We should have protocols which the students must know. Like if this happened this is what you need to do’. (FGD 1, AW)

Students felt that there was a need for better communication and:

‘[*S*]ome direction from the university, I mean dates for next year, sup dates and stuff, and things that affect us directly. And we don’t yet know how next year’s blocks are going to be structured [*as of 14 December 2020*]. So I think some communication from university would be nice’. (FGD 3, DD)

They also felt that staff needed to ‘get training in terms of how they can optimize our teaching and learning’ (SSI 4, LZ) so that they could engage students more effectively using Zoom by using ‘the breakaway room, the little multiple-choice quizzes and the polls’ (SSI 4, LZ). Students wanted clinical staff to help them to ‘focus on the most important issues’ (FGD 2, ME) to ensure good understanding of the topics, and help them to develop an approach to clinical problems rather than trying to cover everything. In addition, students felt that when they went back onto the clinical platform:

‘Doctors should have patience with us and teach us more. Rather than expecting us to know how to do procedure. Next year they should be teaching us procedures and making sure that we catch up’. (FGD 4, SS)

The students felt that the medical school needed to develop a comprehensive catch-up plan to enable them to acquire skills that they did not acquire in 2020. They suggested that:

‘Every discipline has to come up with a plan for students to cover what we were supposed to learn this year, especially with skills and procedures because we were not in the wards, we were not touching patients and when you’re an intern in two years’ time, you will be having to do all of those things. There has to be designated compulsory time for us to actually get into that’. (FGD 2, SM)

Most students were keen to go back: ‘to hospital every day, to get the clinical exposure’ (FGD 4, LG) and to ‘make the blocks face to face with on-site teaching for the blocks, instead of doing the online learning, like we did during lockdown’ (FGD 2, AS). This was because although:

‘[*W*]e learnt a lot of theory, but clinically is what they need to really expose us to next year. Next year, they should just make most of the stuff clinical because we want to be good interns’. (FGD 4, L)

### Grateful that the academic year was salvaged

Despite the challenges and all that had happened, the students were grateful that the academic year had been salvaged and that they were able to complete the academic year and progress to final year:

‘I’m just glad we were able to salvage the year, being able to salvage the year for me is a great positive and we will be going to sixth’. (FGD 3, NB)

‘I’m proud of us for doing that, we’ve actually finished the academic year in six months. I mean, that’s very, wow’. (FGD 4, SS)

While grateful that they had been able to salvage the year and had progressed, they were sympathetic to students who had failed, as:

‘[*T*]hose getting left behind, (was) not because they are bad students or not because they’re just disinterested, but because they haven’t been able to engage with the material like others have’. (FGD 1, DC)

## Discussion

The results will be discussed under the following three headings: stress and mental health, developing coping strategies and building resilience, and teaching and learning.

The COVID-19 pandemic has had a major impact on all aspects of life, and the various lockdowns have led to disruptions in education, social isolation, loss of earning potential, anxiety and fear about the future.^1^ Students have had to adjust to online and blended learning, and academic staff have had to learn how to use the various technologies and adjust their programmes to deal with the challenges arising from the pandemic. Much has been written on the pedagogy of online teaching and learning, opportunities for enhancing distance learning and simulated teaching of skills via videos.^3^ However, little has been written in South Africa on the learning experiences during this time of crises for undergraduate students in general, and medical students in particular due to their extensive practical training requirements.

### Stress and mental health

Consistent with the finding of this study, high levels of anxiety and stress during lockdown have been reported among medical students from a number of studies around the world.^4,5,6,7,8^ These heightened levels of stress are due to multiple factors, such as the loss of personal freedom, lack of social support and loneliness due to social isolation, frustration, the unpredictable nature of the disease, uncertainty about the future, curricular disruptions, academic delays, fear of COVID-19 infection, loss of loved ones due to COVID-19, poor communication from authorities, financial uncertainty when family members lose their jobs, as well as exposure to excessive social media.^4,5,7,8,9,10^ Natural and environmental social disasters are almost always accompanied by increases in depression, post-traumatic stress disorder, as well as mental and behavioural disorders,^6^ making it understandable as to why students reported high levels of stress. The UKZN does has an active student support programme which seeks to provide psychological support to all students. However, student responses indicate that from their perspective, university related support in 2020 was not effective. This needs further investigation to ensure that the university is able to provide proactive, appropriate psychological support to health science students who have returned to the clinical platform.

Lasheras et al. suggested that medical students may be more susceptible to developing high levels of anxiety due to their type A personalities, challenging academic training and the fact that, in general, they are less likely to seek support when affected by psychological stressors.^7^ In an assessment of anxiety among 852 1st to 4th year medical students across 24 training institutions from June 2020 to August 2020 in the United States (US), 66.1% reported anxiety symptoms (mild: 34.98%, moderate: 19.25% or severe anxiety: 11.85%),^4^ with stress highest in the more senior years. This is consistent with a study at UKZN,^8^ which reported a 60.0% prevalence of mental stress among 5th year medical students.

These findings differ, however, from a meta-analysis of eight international papers published in 2020 which estimated the prevalence of anxiety among medical students to be only 28%.^7^ The authors of the meta- analysis concluded that the lower levels of anxiety may be due to access to relevant information, high levels of resilience and satisfaction with online learning among medical students.^7^ This requires further research, but suggests the importance of providing accurate, reliable, up-to-date information to students about the pandemic and its impact on their teaching and assessment, building resilience and ensuring that they have access to quality appropriate online learning material.

### Developing coping strategies and building resilience

Although the word resilience was not used, the participants talked about the need to develop strategies to help them cope during the lockdown(s) and to deal with the academic pressures when they returned to full-time study. Resilience has been described by Robertson et al. as a positive adaptation and the development of personal resources, growth and hardiness to withstand adversity, as well as the ability to bounce back from challenging situations.^11^ or ‘a dynamic process encompassing positive adaptation within the context of significant adversity’.^12^ Without a doubt, the COVID-19 pandemic highlighted the need for resilience for both students and staff.

Greater resilience is associated with many of the following: participating in regular physical activity, achieving life balance which includes participating in enjoyable leisure activities and spirituality, developing self-efficacy and coping strategies, building positive and nurturing relationships, becoming more reflective and developing emotional insight.^10,11,12^ Those who show greater resilience adapt positively to challenging and changing circumstances and are able to maintain a sense of personal fulfilment in these circumstances. Other qualities seen in resilient people include resourcefulness, self-confidence, curiousness, self-discipline, level-headedness, flexibility, as well as emotional stamina and problem-solving^11,12^ qualities which would help medical students to thrive.

Although there is some teaching on time management in the junior years at the medical school, resilience building as a strategy for students to thrive in challenging circumstances is not covered and needs to be incorporated into the medical curriculum. This could be done by helping junior students to develop a wellness plan that could be reviewed and updated each year. Such a wellness plan should cover issues such as mental well-being, physical well-being, academic goals and progress, and activities outside of the home or residence, particularly those that help give meaning and a sense of purpose. To be effective, such a wellness plan would need to be developed, submitted, marked, regularly reviewed and be part of a support programme that links small groups of students to academic staff who could track their progress and provide support. These huddle/support groups could promote reflection, discussion on issues faced, fears and expectations within the context of supportive and ongoing relationships that have been shown to help in the development of resilience.^10^

### Teaching and learning

Shortened blended blocks were a temporary, emergency solution to salvage the 2020 academic year. Although appreciative of the effort and work of the academic staff to ensure that the majority of students were able to progress, it is important to acknowledge what was lost in teaching, skills development and clinical competence. Careful thought needs to be given to what additional support can be provided to students who recognise their deficiencies and who want to be competent. This is an ongoing challenge, as South Africa is currently (December 2021) entering the 4th wave of the COVID-19 and most teaching has not returned to normal. However, as all students in their clinical years should have been vaccinated by the time the new academic year starts in January 2022, it is essential that medical students return to the clinical platform where they can learn around patients, as this is the context in which competent clinicians are trained. Without doubt there is an important place for online teaching and training, as it can be used to cover the theoretical content. However, this form of training can never replace on-site, hands-on, apprenticeship type learning, which is essential to produce competent clinicians.

Students want to be competent clinicians and the academic staff need to ensure that the teaching and training provided develops these skills. The academic staff must ensure that programmes are designed to meet clear aims and objectives, are delivered in the most effective manner, and assessed fairly. However, students highlighted the large volume of content that they were expected to cover, how they responded and the fact that for many they simply learned for the exams (exam competent vs. clinically competent). The COVID-19 pandemic has given academic staff the opportunity to critically re-evaluate the medical curriculum, to determine what is core, whether what they are teaching is useful, essential or simply a bonus,^13^ as well as how they teach and assess what is being taught. As highlighted by the students, the academic staff need to focus on what is essential, and to streamline the curriculum to enable students to integrate and apply information, rather than try and remember every fact, many of which can be looked up.^13^ Streamlining the curriculum will leave time for other important extra-curricular activities and self-care.

Online platforms has been shown to be excellent for theoretical and for simulated teaching^14,15,16^ and a flipped classroom approach with discussion forums promotes student engagement in the teaching process.^3,14,17,18^ There are a range of online platforms and software applications that promote teaching and learning, and allows for synchronous and asynchronous teaching.^14,18^ Assignments via online platforms have also been effective for promoting critical thinking and summative assessments, and have been used for OSCEs, where the examination has tried to recreate a clinical environment online.^14^

However, online learning is inadequate for some aspects of socially collaborative learning/social aspects associated with learning (collaboration, peer learning, student cohesion, group identity, socially mediated development of applied knowledge, learning by fostering interaction between learners, and between learners and facilitator)^3^ and is unable to provide practical hands-on learning.^14,19^ A 2020 study among 440 final year medical students at 32 medical schools across the United Kingdom (UK) reported that 59% of the final year students felt inadequately prepared for hospital work^17^ as a result of the lockdown measures and the impact that this had on their medical education.

The COVID-19 pandemic has provided an opportunity for staff to be innovative about assessments and how these could be run. The UKZN family medicine block introduced an online OSCE that enabled consistent student assessment in an environment that was safe for staff and students. Online OSCEs have been successfully used elsewhere.^14^ Similarly, the Colleges of Medicine of South Africa have introduced video-based assessments as well as evaluation of online consultations. While students appreciated the innovation, it is important that these innovations be rigorously evaluated to determine their reliability as assessment tools.

With the need to return to clinical teaching to ensure competent graduates, academic departments must not fall back into simply doing what they did prior to the pandemic, but must use this opportunity to develop a blended approach that incorporates the best of online and on-site teaching to maximally enhance student teaching and learning.^15^

## Conclusion and recommendations

The COVID-19 pandemic and the resultant lockdown(s) have been a stressful and difficult/challenging time for everyone, but particularly for students, more so than the academic staff may have realised. The disruptions have impacted on teaching and learning and the perceptions of competency among students. When planning for the future, the academic staff must be clear about the possibilities and limitations of online learning, and the need to use relevant teaching tools to actively engage with students. Although there is a vast amount of material to cover, departments need to use the opportunity provided by the pandemic to take cognisance of the teaching time available, help students focus on the important issues and how to develop an approach to particular issues rather than having students just memorise large quantities of information. Clear communication is essential in a crisis, and while the students can accept uncertainty, there needs to be consistent, honest and timely messages.^13^ It is clear from the feedback that UKZN initially failed in this regard, with a need in the future for the university to communicate clearly to ensure students understand the challenges faced by the university as well as what is expected.

In addition, when planning for the future, proactive strategies to help students develop greater resilience need to be developed, implemented, monitored and evaluated to enable them to show fortitude and bounce back from adversity, not only in their current situations, but as a life skill. Proactive programmes that can identify students at risk of mental health challenges, and that can provide appropriate support when and where needed, should be developed as a priority.

## Limitations

While this is a qualitative study with all the associated limitations, it does capture the opinions of medical students and their perspectives of learning in 2020, with many of the themes identified possibly be applicable to other students in South Africa who were engaged in online and blended learning.

One of the main advantages of FGDs over SSIs is the interaction between participants. Interviews conducted over Zoom made this research possible but also had limitations, being difficult for participants to interject and comment on issues being presented as only one person can be heard at a time, which limited the active interaction between participants. Although there is a huge research potential with Zoom, it is important to understand its limitations (cannot speak over, difficult to read body language etc.), highlighting the need for further research to understand how to incorporate online platforms into our research activities.
